# Isolation and Identification of a Genotype C Bovine Parainfluenza Virus Type 3 and Its Pathogenicity in Albino Guinea Pigs

**DOI:** 10.1155/2023/8854528

**Published:** 2023-11-23

**Authors:** Yu Han, Fathalrhman Eisa Addoma Adam, Riteng Zhang, Yan Gao, Jianlin Lei, Kejia Lu, Xinglong Wang, Sa Xiao, Haijin Liu, Zengqi Yang

**Affiliations:** ^1^College of Veterinary Medicine, Northwest A&F University, Yangling, Shaanxi, China; ^2^Yulin Animal Husbandry and Veterinary Service Center, Yulin, China

## Abstract

Bovine parainfluenza virus type 3 (BPIV3) is a significant pathogen in cattle, causing a range of respiratory symptoms from mild to severe. In this study, we isolated a BPIV3 (BPIV3/SX/2021) strain using Madin-Darby bovine kidney cells from a lung tissue sample obtained in Shaanxi province. The full length of the BPIV3/SX/2021 strain is 15,474 nt, encoding six structural and three accessory proteins. A phylogenetic analysis revealed that SX2021strain belongs to BPIV3 genotype C, and the isolate shares the highest sequence homology with the other genotype C strains isolated in China. A genetic comparison of the BPIV3c strain isolated in China showed that some amino acid residues have mutations in the F and HN genes that affect the protein structure. Animal challenge experiments showed that experimental infection of guinea pigs with the BPIV3c strain SX2021 displayed depression and dyspnea clinical signs. Histopathological analysis of the lung and trachea showed that morphological changes consisted of lesions of bronchial epithelial cell shedding, alveoli septa thickening, focal fiber hyperplasia, and macrophage infiltration. The virus load analysis indicated that the SX2021 strain displayed a higher replication level in the lungs and trachea. These results provide strong evidence that the evolution of BPIV3, and the isolated BPIV3 strain SX2021 provide useful data to determine the pathogenesis of the virus.

## 1. Introduction

Bovine respiratory disease complex (BRDC) is a severe illness impacting feedlot cattle around the world [1, 2]. Bovine parainfluenza virus type 3 (BPIV3) is one of the most significant viral pathogens in BRDC [2]. BPIV3 exhibits significant virulence across all cattle age groups, with observed clinical signs including coughing, pyrexia, anorexia, nasal and ocular discharges, dyspnea, and occasionally diarrhea [3]. BPIV3 is an enveloped, nonsegmented negative-strand RNA virus, that belongs to the genus *Respirovirus* in the family *Paramyxovirdae* [4]. In 1959, the first BPIV3 was isolated and detected in cases of “shipping fever” in the USA [5, 6]. Up to now, BPIV3 has occurred around the world.

BPIV3 has been classified into three genotypes including genotype A, genotype B, and genotype C. Among these three types, genotype A of BPIV3 strains has been isolated worldwide, including in Australia, the USA, China, Argentina, and Japan [7]. Genotype B is primarily found in Australian cattle and water buffaloes in Argentina [2, 8]. In 2008, four BPIV3 strains were isolated in China, and nucleotide phylogenetic analysis suggested the emergence of a potentially new genotype, marking the first identification of genotype C in China [9]. Since then, genotype C has been identified in South Korea, Japan, Argentina, and the United States [10–12]. High seropositivity rates for BPIV3 in dairy cattle indicate widespread infections, prompting efforts to prevent and control BRDC to mitigate production losses in the livestock industry.

The virions of BPIV3 are pleomorphic and have a diameter of 150–250 nm. The genome organization resembles that of other paramyxoviruses; the complete genome size is about 15 kb and encodes six structural proteins [13]. The P gene of BPIV3 contains three unstructural proteins, including C, V, and D proteins. The V and D proteins are translated from two downstream overlapping ORFs by two mRNA editing sites, while the C protein is translated from an alternative translation initiation site, resulting in a different reading frame [14]. The 5′ and 3′ noncoding region of the BPIV3 genome constituted the trailer and leader sequence, and the genome structures of two genes were flanked by gene start (GS) and gene end (GE) sequences.

The fusion protein is a membrane protein, that mediates viral entry into host cells by fusing the viral with the cell membrane [13, 15]. The HN protein is a modular protein with discrete regions responsible for receptor attachment, tissue tropism, and triggering the fusion activity of the protein [13, 15]. Numerous studies claim that the F and HN proteins of paramyxovirus play a vital role in viral factors, including viral replication and tissue tropism, which influence viral virulence and the ability to evade the host immune response [16].

In recent years, an increasing number of BPIV3-induced respiratory disease outbreaks in cattle have led to substantial economic losses. To date, only limited studies of BPIV3 infection in animals have been reported. In this study, a BPIV3 strain, BPIV3-SX-2021, was isolated and characterized. Animal challenge experiments with albino guinea pigs showed that viral infection could induce respiratory symptoms. These data provide useful data in studying the pathogenesis of BPIV3.

## 2. Materials and Methods

### 2.1. Clinical Samples Collected and PCR Detection

In November 2021, an acute outbreak of BRDC occurred on a cattle farm in Shaanxi province. A lung tissue sample from sick cattle was collected and stored at −80°C.

The viral RNA and DNA were extracted using the PureLink viral RNA and DNA Mini Kit (Invitrogen) according to the manufacturer's instructions and reverse transcribed as cDNA using a StarScript II RT Kit (Genstar). Molecular assays were used to identify the viruses that might be associated with similar clinical signs, including BPIV3, (Bovine respiratory syncytial virus) BRSV, and (Bovine herpesvirus1) BHV-1. The primers are listed in Table 1.

### 2.2. Cells, Reagents, and Virus Isolation

Madin-Darby bovine kidney (MDBK) cells were cultured in Dulbecco's modified Eagle's medium (DMEM, GIBCO) supplemented with 10% fetal bovine serum (FBS) at 37°C with 5% CO_2_. The lung tissue sample was sterilized by passage through 0.22 *μ*m inoculated into MDBK cells cultured in 60 mm cell culture plates. The supernatants were harvested until a cytopathic effect (CPE) was observed.

### 2.3. Virus Identification

#### 2.3.1. RT-PCR Analysis

BPIV3 strain SX-2021 was harvested after the fifth passage for extracting viral RNA by TRIzol reagent (Takara) and reverse transcribed as cDNA using a StarScript II RT Kit (Genstar, Beijing, China). The amplification of cDNA by PCR used a primer (BPIV3-F/R). PCR products were detected by agarose gel electrophoresis and sequenced.

#### 2.3.2. Indirect Immunofluorescence Assay

MDBK cells were seeded in a 24-well plate and were infected with BPIV3-SX-2021 at an multiplicity of infection (MOI) of 0.1 and mock infected. At 24 hr, cell monolayers were fixed with 4% paraformaldehyde for 30 min at room temperature and blocked with PBS containing 1% bovine serum albumin for 30 min. The cells were then permeabilized with 0.1% Triton X-100 for 5 min. After 1 hr of incubation with primary anti-NP (diluted 1 : 200), cell monolayers were washed with PBS three times and further incubated for 1 hr with secondary antibody, Alexa Fluor 488-labeled Goat Anti-Mouse IgG (H + L, Yeasen, Shanghai, CN). Cells were examined under a fluorescent microscope after being washed three times, and images were captured using the EVOS FL Cell Imaging System (IX73; OLYMPUS, Tokyo, Japan).

#### 2.3.3. Western Blotting Analysis

The MDBK cells were seeded in a 24-well plate and infected with BPIV3/SX/2021 at an MOI of 0.1. At 24 hr, cell lysates were harvested with 80 *μ*L/well Lysis Buffer containing 1% PMSF. After centrifugation at 15,000 g for 15 min, cell debris was removed. The cell lysate was mixed with 5x loading buffer (Bio-Rad, Hercules, CA) and denatured at 95°C for 10 min. Proteins were transferred onto PVDF and the membrane was blocked with 5% skim milk at room temperature for 3 hr. To detect the expression of BPIV3 NP, the membrane was incubated with primary antibodies at 4°C, including a pAb against NP (diluted 1 : 1,000). After 12 hr of incubation, the membrane was washed with TBST and then probed with secondary antibodies, HRP-labeled Goat Anti-Mouse IgG (H + L, Beyotime, Beijing, CN). The target proteins were exposed using BeyoECL Plus (Beyotime, Beijing, CN).

#### 2.3.4. Plaque Assays

MDBK cells were seeded in 24-well plates, Serial 10-fold dilutions were made by mixing BPIV3-SX-2021 with DMEM. After adsorption at 37°C for 1 hr, the supernatants were removed and covered with medium containing 1% low melting point agarose. After 5 days of incubation at 37°C, the cells were fixed in 4% paraformaldehyde and then stained with 1% crystal violet.

#### 2.3.5. The TCID_50_ Assay and Virus Growth Kinetics

MDBK cells in a 6-well plate were infected with BPIV3-SX-2021 at an MOI of 0.1. The plates were incubated for 1 hr at 37°C, and the cells were washed with PBS and covered with DMEM supplemented with 2% FBS. Cell culture supernatants were harvested at different time points. The viral titers of the supernatants were measured by titration on MDBK cells, and results were reported by the 50% tissue culture infective dose (TCID_50_) assay.

### 2.4. Whole Genome Sequence Analysis

To obtain the whole genome sequence of the BPIV3/SX/2021 strain, the viral RNA of the SX/2021 strain was extracted using a TRIzol reagent. The whole genome sequence was conducted by next-generation sequencing at the Shanghai Tanpu Biotechnology Co., Ltd., (Shanghai, China). Sequence data were analyzed using Snap Gene software.

### 2.5. Phylogenetic Analysis

For phylogenetic analysis, the whole genome sequence of PIV3 reference strains was downloaded from GenBank as a reference. These sequences were used to generate a neighbor-joining phylogenetic tree with MEGAX software, using the *p*-distance model and 1,000 bootstrap replicates. The phylogenetic tree was annotated with the Interactive Tree of Life software, an online tool for the display and annotation of phylogenetic trees.

### 2.6. Molecular Modeling and Analysis of the F and HN Domains of BPIV3

Some amino acid mutations in the F and HN proteins of BPIV3-SX-2021 were aligned with the sequences of three genotype C virulent strains (XJA, NX49, SD0835) using MEGAX software. 3D models of F and HN proteins were constructed by alphafold2, and the amino acids that affect the protein structure were marked with different colors.

### 2.7. Animal Study

The guinea pigs were performed according to protocols approved for the handling of animals and experimentally by the guidelines for caring for laboratory animals of Northwest A&F University (approval number: NWAFUSM20210-11). Twenty guinea pigs were divided into two groups: Group 1 guinea pigs were infected with BPIV3-SX-2021 and Group 2 guinea pigs were mock infected. Every guinea pig was intranasally inoculated with 200 *µ*L of the virus (or culture medium) at a dose of 2 × 10^7^ TCID_50_/mL. Two guinea pigs in every group were euthanized at 1, 3, 5, 7, and 14 days postinoculation; the heart, lung, liver, kidney, spleen, and trachea were separated into two parts. One part of the tissue samples was used for testing the viral load in different tissues by virus isolation and titration. The viral titers were measured via a TCID_50_ in MDBK cells using the Reed and Muench method. The other part was fixed in 10% formalin for 48 hr at room temperature, and the fixed tissues were routinely embedded in paraffin wax, sectioned, and stained with hematoxylin–eosin. The samples were examined for lesions using light microscopy. To test the virus's neutralization, blood samples were collected by cardiac puncture. Briefly, the serum samples were heat-inactivated at 56°C for 30 min and twofold serially diluted in DMEM in a 96-well plate. The mixture was mixed with 100 TCID_50_ virus and incubated at 37°C for 1 hr and the serum–virus mixture was inoculated into MDBK cells. The neutralization titer was considered the highest dilution of serum which resulted in a reduction of CPE by at least 50%.

## 3. Results

### 3.1. Virus Isolation and Characteristics in Cattle with Clinical Signs

**Figure 1:**
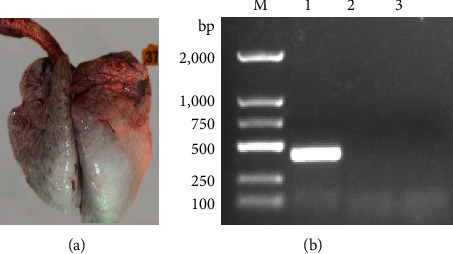
The lung tissue of cattle with respiratory disease and PCR detection. (a) A lung tissue sample from sick cattle. (b) PCR detection of bovine parainfluenza virus type 3 (BPIV3), bovine alphaherpesvirus 1 (BHV-1), and bovine respiratory syncytial virus (BRSV).

**Figure 2:**
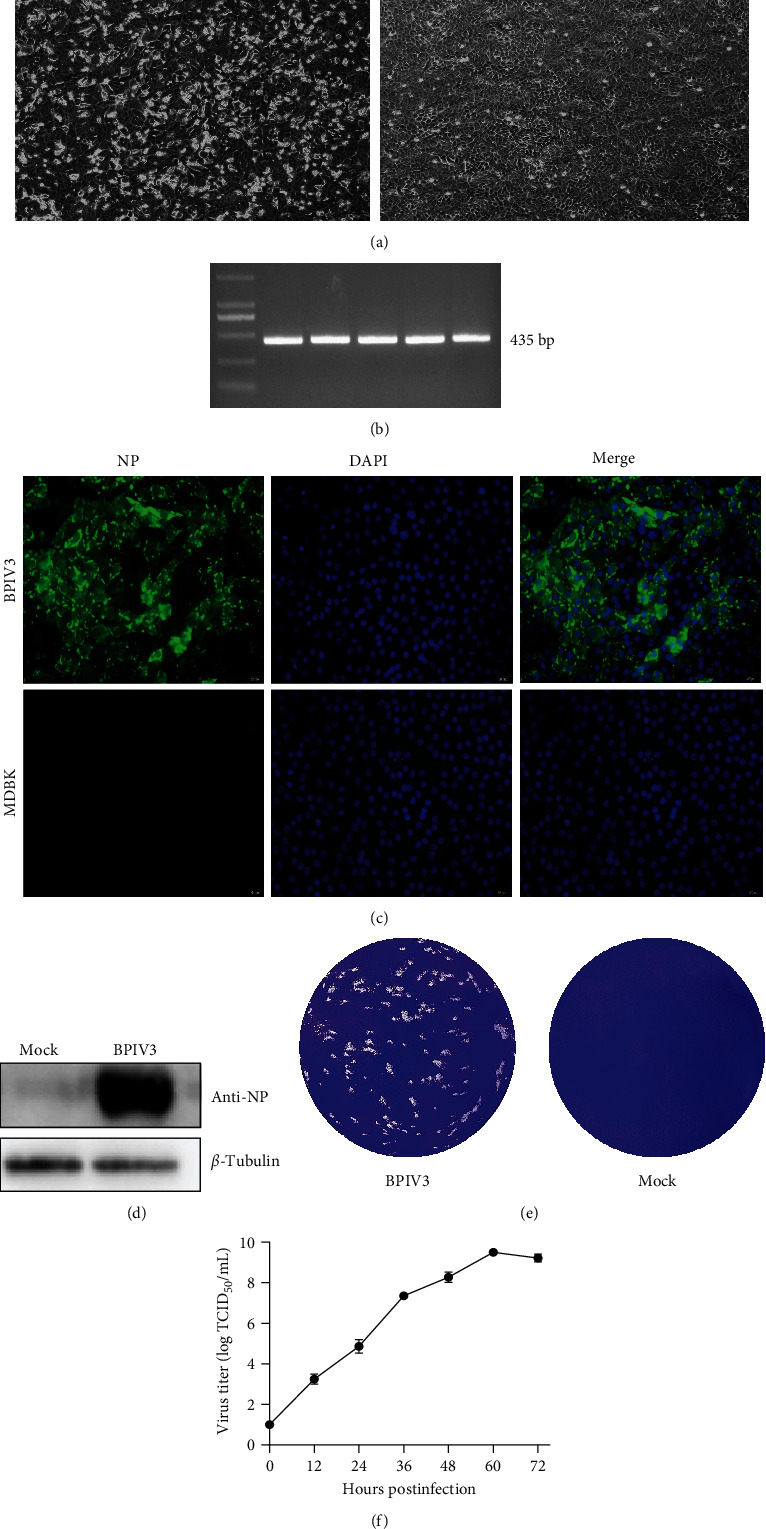
Isolation and identification of the BPIV3 strain BPIV3-SX-2021. (a) Cytopathic effects (CPE) of the BPIV3-SX-2021 isolates in infected MDBK cells, such as shrinkage, rounding, and nonadherence, were observed in MDBK cells infected with BPIV3-SX-2021 at 48 h.p.i. No CPE was observed in uninfected cells. (b) RT-PCR analyses of BPIV3-SX-2021 at *F*1, *F*2, *F*3, *F*4, and *F*5 using the BPIV-F/R. (c) Indirect immunofluorescence assay of BPIV3-SX-2021-infected MDBK cells using the anti-NP antibody. (d) The viral protein NP was detected with western blotting using an anti-NP Polyclonal antibody. (e) Plaque morphology of the BPIV3-SX-2021 in MDBK cells at 96 h.p.i. (f) The growth curves for the viruses and viral titers were determined with a TCID_50_ assay. The data represent the mean ± SD for three independent experiments.

To determine the pathogen of this outbreak, a lung tissue sample was used to detect the pathogens causing BRDC diseases by RT-PCR assays. However, in the lung tissue sample, no corresponding nucleotide fragments were observed for BRSV and BHV-1 and positive for BPIV3 (Figures 1(a) and 1(b)). To obtain the BPIV3 strain, the samples were further cultured in MDBK cells to isolate the virus, and the CPEs were observed at 36 h.p.i (Figure 2(a)). The BPIV-F/R-based RT-PCR showed that the 435-bp-specific product was successfully amplified from the BPIV3 strain after the fifth passage (Figure 2(b)). To further identify the strain, the indirect immunofluorescence assay result showed that fluorescence reflected the expression of the viral NP in the cytoplasmic of the infected cells (Figure 2(c)). Western blotting also confirmed the expression of BPIV3 NP in the infected cells, and the molecular weight of the NP protein was about 69 kDa (Figure 2(d)). MDBK cells infected with the virus could form distinct plaques at 5 days postinfection (d.p.i.; Figure 2(e)). The virus titer was 10^9.5^ TCID_50_/mL at 60 h.p.i. Based on the results of virus isolation and identification, the newly isolated virus strain was named BPIV3-SX-2021.

### 3.2. Characterization of the Complete Genome Sequencing Analysis

The full-length genomic of BPIV3-SX-2021 was 15,474 nt, which conforms to “the rule of six” with 36.7% GC content. BPIV3 encodes six structural proteins and three accessory proteins. The 3′ and 5′ ends of the SX2021 strain genome comprised the leader and trailer regions, respectively. The virus displayed a similar genome structure to other paramyxoviruses in that every structural protein is flanked by a conserved GS sequence and GE sequence. The GS, GE, and intergenic regions sequences of the SX2021 strain are shown in Table 2. The complete genome sequence of strain BPIV3-SX-2021 has been deposited in GenBank (GenBank accession no. ON804787).

### 3.3. Phylogenetic Analysis of Strain SX2021

**Figure 3:**
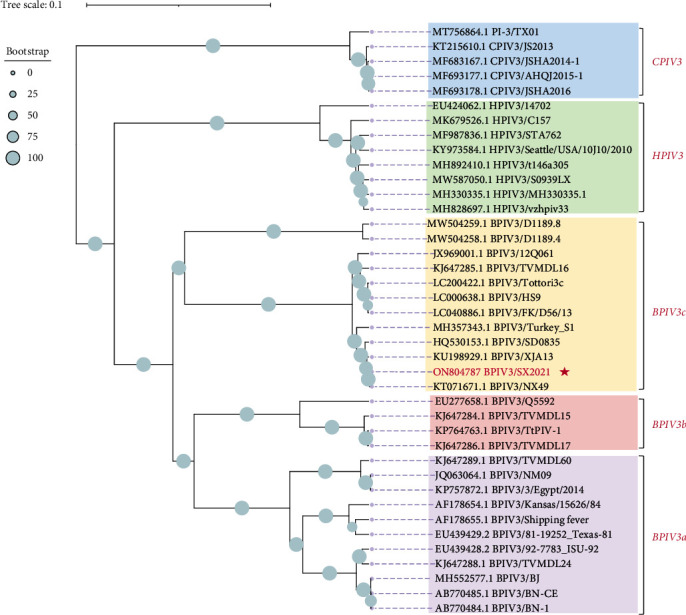
Phylogenetic analysis based on the full-length genome of BPIV3-SX-2021 and reference PIV3 strains. A phylogenetic tree was constructed using the neighbor-joining method in the MEGA version X program, with bootstrap validation using 1,000 replications. Different hosts and genotypes are shown in distinct colors. The virus used in this study was colored red, and that strain belongs to genotype C.

A phylogenetic tree was constructed with the whole genome sequences of PIV3. Overall, the phylogenetic tree showed that the 27 available strains isolated from cattle can be divided into three groups (Group A–C) and that SX-2021strain belongs to genotype C. BPIV3-SX-2021 shared the highest homology with the other genotype C strains isolated from China (Figure 3).

### 3.4. Molecular Modeling and Analysis of the F and HN

**Figure 4:**
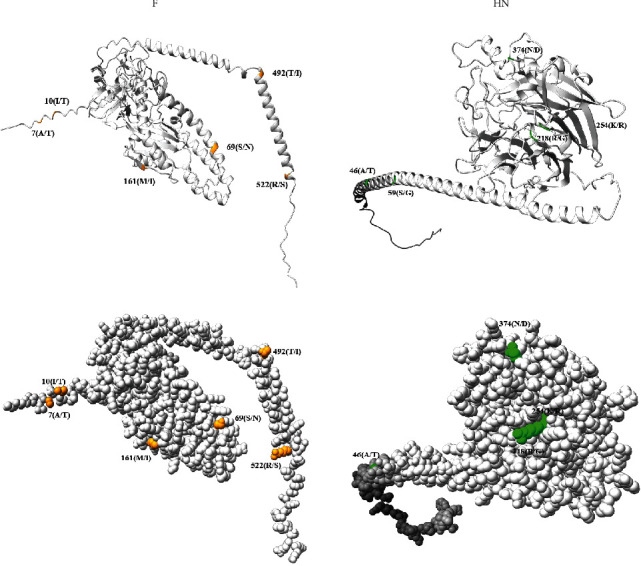
Localization of amino acid residues. 3D structural models were constructed of the F and HN proteins online (https://alphafold.ebi.ac.uk) using ChimeraX software. The amino acid residues are labeled in orange (F) and green (HN).

The F and HN proteins were related to virus immunogenicity and virulence in the family paramyxovirdae. To understand the mutations at the aa sites of BIV3-SX-2021, three virulent BPIV3 strains (XJA13, NX49, and SD0835) were selected, and the sequence alignment results showed that several amino acid residues in the F and HN proteins were altered (Table 3). In the 3D models of the F and HN protein, these amino acid mutations were marked in yellow (F) and green (HN) using the Chimera X software (Figure 4). The locations of the amino acid residues on each protein might affect the protein structure and the interaction between the viral protein and the cellular receptors. These amino acid residue mutations in the BPIV3 isolate should be studied with experiments in the future.

### 3.5. Pathogenicity of the Isolate in Guinea Pigs

**Figure 5:**
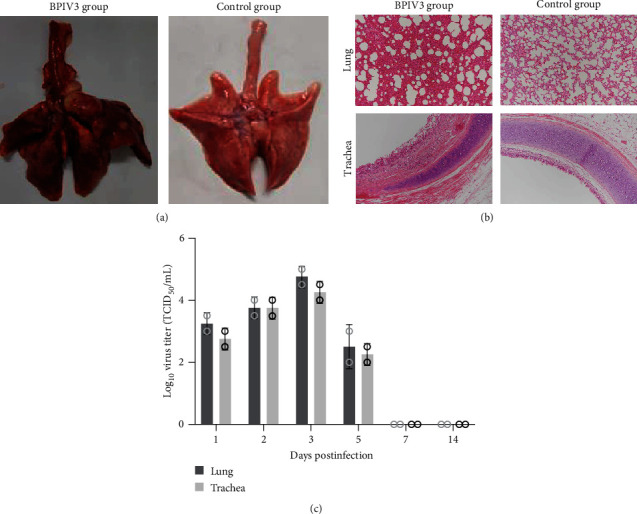
The animal challenge of guinea pigs with strain BPIV3-SX-2021. (a) Macroscopic appearance of lungs from guinea pigs. Almost complete consolidation and atelectasis of lung lobes from one guinea pig at 3 days infected with BPIV3 strain SX-2021. A normal control lung from a mock-inoculated guinea pig. (b) The lung and trachea tissue of guinea pigs infected with BPIV3-SX-2021 after hematoxylin and eosin (H&E) staining (100x magnification). (c) Viral load of SX2021 in guinea pigs infected with SX2021.

The guinea pigs of the mock group were clinically normal, and the guinea pigs of the infection group began exhibiting clinical signs at 3–7 days. The clinical signs consisted of depression and dyspnea. At necropsy, the gross pneumonic lesions in infected guinea pigs consisted of lung lobes that were slightly depressed and dark red. Other organs showed no clinical symptoms (Figure 5(a)).

Histopathological analysis showed that the lung and trachea in the infection group showed obvious histopathological changes. The histopathological analysis of the lung showed bronchial epithelial cell shedding, thickening of the alveoli septa, focal fiber hyperplasia, and macrophage infiltration. In addition, the tissues of the trachea had histological modifications such as lymphocytic infiltration and mucosal epithelial cell loss. All the guinea pigs in the control group had healthy lungs and tracheas (Figure 5(b)). The heart, liver, spleen, and kidney of the infected and mock groups did not show morphological changes (Data not shown).

Virus isolation and titration measured the viral loads from 0.2 g of the lung and trachea tissues. The virus load results showed that the lungs and trachea tissues were positive for 1–5 days. The highest viral load in the lung and trachea was 10^5^ TCID_50_/mL and 10^4.5^ TCID_50_/mL at 5 days, respectively. No virus was tested in the control guinea pigs (Figure 5(c)).

Blood samples were taken from the guinea pigs infected with SX2021, and the viral neutralization titer was tested. The guinea pigs infected with BPIV3 strain SX2021 had virus neutralization antibodies with a titer of 1 : 4 at 7 d.p.i, and the titer reached 1 : 16 at 14 d.p.i. The virus neutralization titer remained negative in the mock group.

## 4. Discussion

BPIV3 is the most significant bovine respiratory disease that affects livestock industries worldwide, particularly in large cattle-producing countries like China, the USA, and Australia [17, 18]. Since the first report of BPIV3 in early 1959s, outbreaks have constantly occurred, and numerous BPIV3 strains have been identified [5, 6]. In 2008, seven isolates of BPIV3 were reported in Australia, and sequence analyses showed that four of the seven strains were distinct from the other three isolates, indicating that this viral species can be classified as genotype B [2]. In the same year, four BPIV3 strains were different from the previously reported and might be a potentially new genotype, which was tentatively classified as genotype C [9]. Afterward, genotype C of BPIV3 has also been identified in South Korea, Argentina, Japan, and the USA, of which strains in genotype A and C became predominant among cattle. In Japan, 73 BPIV3 strains were isolated between 2002 and 2019, and phylogenetic analysis showed that the isolates were classified into two genotypes, BPIV3a (49%) and BPIV3c (51%) [19]. Several studies have shown that only a limited number of type A and type C strains were isolated in China, and genotype C has caused outbreaks in China [7, 9]. Given the serious impact of BPIV3 on livestock industries, we collected BPIV3-positive cattle samples in Shaanxi province to isolate and identify the causative agent. BPIV3 strain SX2021 was successfully isolated, and phylogenetic analysis showed that the SX2021 strain classified genotype C and shares the highest homology with other genotype C strains isolated from China. Recently, the first type B Strain of BPIV3 was isolated and identified, thus enriching our knowledge of BPIV3 genotypes in China [20]. All these indicate that the BPIV3 strains circulating in China are genetically diverse and challenging the current biological control measures.

Several studies showed that F and HN proteins are glycoproteins that play a crucial role in the determinants of virulence and immunogenicity in the paramyxovirdae family [16]. In this study, to understand the putative virulence-related viral proteins and the key amino acid of SX2021, three genotype C strains isolated from China were selected, and the sequence alignment results showed several amino acid residues in the F and HN proteins. These locations of amino acid residues might affect the protein structure and the interaction between the viral protein and cellular receptors. However, animal challenge showed that strain SX2021 is highly contagious. Thus, the mutation of these amino residues in the F and HN proteins may be uncorrelated with their virulence. But the 3D models of these proteins showed that the amino acid residues affect the protein structures. Unfortunately, we still have insufficient evidence to demonstrate the association between these amino residues and their immunogenicity. The roles of these amino acid residues in the immunogenicity of the BPIV3 isolate should be studied with experiments in the future.

Several studies have shown that PIV3 is often detected in humans or animals with respiratory symptoms, especially in the lung and trachea, including cattle, pigs, goats, sheep, and buffalo [21–23]. However, it was not easy to develop an animal infection model in cattle. Laboratory animal models were usually used in studying the pathogenesis. In an earlier report, BPIV3 infection in Balb/c mice and albino pigs was conducted and reported. Intranasal inoculation of BPIV3c in Balb/c mice did not cause signs of disease or gross pathological alternations [22]. In addition, guinea pigs who received an intranasal BPIV3c inoculation showed some signs of illness and developed some gross pneumonic lesions, which were similar to those seen in calves that had an experimental BPIV3 infection [21]. Thus, guinea pigs as laboratory animals were used to study the pathogenesis of BPIV3. In this study, the guinea pigs infected with the SX2021 strain showed clinical signs in the lung and trachea after 3 days. The histological sections showed a morphological change in the lung and trachea tissues, and a viral load was measured in the lung and trachea tissues, showing that guinea pigs were permissive for SX2021 replication during the first to seventh days. VN antibodies against SX2021 were detected 7–14 days postinoculation, showing that VN antibodies could provoke specific immune responses. Therefore, a laboratory animal model was constructed that provided a useful tool to further experiment with pathogenicity.

In this study, the BPIV3/SX/2021 strain was isolated from the lung tissues of cattle that showed clinical signs of pneumonia. Whole-genome sequence analysis showed that the SX/2021 strain belongs to genotype C. Compared with the three strains of this virus, the F and HN proteins of strain SX2021 have a structural mutation. A pathogenicity analysis confirmed that the SX/2021 strain is highly virulent in guinea pigs that showed obvious respiratory disease symptoms. In summary, we isolated and characterized a new pathogen that provides useful data for studying the pathogenicity and genetic evolution of BPIV3.

## Figures and Tables

**Table 1:** The sequence of primers used in this study.

Primers	Sequences (5′–3′)
BPIV-F	GGATGTTTGGAAGTGATCTTGAGT
BPIV-R	TGTATTAAAGAATGAAGCAAGGCCTG
BHV-F	TACGCGGCCATTACAAACCAG
BHV-R	AGCACACGTTTTTGCGCTTG
BSRV-F	ATGGCTCTTAGCAAGGTCAA
BSRV-R	TCCATTTCTGCTTGTACGCT

**Table 2:** Gene star, gene end, intergenic sequences, and predicted proteins of SX2021 strain.

Gene region	Size (nt)	Gene start	ORF positions	Gene stop	IGR
Leader UTR	110	AGGATTAAAG 56-65	111-1658	AAATAAGAAAAA 1690-1701	CTT
NP	1,548				
N-P UTR	125	AGGATTAACG 1705-1714	1784-3586	AATCAAGAAAAA 3700-3711	CTT
(P)	1,803				
P-M UTR	160	AGGACAAAAG 3715-3724	3747-4802	CAGAATCAAAAA 4852-4863	CTT
(M)	1,056				
M-F UTR	293	AGGATTAAAG 4867-4876	5096-6718	AGGTATAAAAAA 6742-6753	CTT
(F)	1,623				
F-HN UTR	99	AGGAACAAAG 6757-6766	6818-8536	AAAAATAAAAAA 8621-8632	CTT
(HN)	1,719				
HN-L UTR	121	AGGAGAAAAG 8636-8645	8658-15359	GTAAATAGTGTA 15372-15383	CTT
(L)	6,702				
Tailer UTR	115				

**Table 3:** Some amino acid residues in the F and HN protein.

	F						HN				
The sites of amino acid	7	10	69	161	492	522	46	59	218	254	374
SX2021	A	I	S	M	T	R	A	S	R	K	N
XJA13	T	I	N	I	I	R	A	S	G	R	N
NX49	T	I	S	I	I	R	A	S	G	R	D
SD0835	T	T	N	I	I	S	T	G	G	K	N

## Data Availability

The whole genome sequence of the BPIV3/SX/2021 strain is openly available in the NCBI GenBank Accession number ON804787.
